# Autophagy is associated with a robust specific transcriptional signature in breast cancer subtypes

**DOI:** 10.18632/genesandcancer.208

**Published:** 2020-10-06

**Authors:** Céline Grandvallet, Jean Paul Feugeas, Franck Monnien, Gilles Despouy, Perez Valérie, Guittaut Michaël, Eric Hervouet, Paul Peixoto

**Affiliations:** ^1^ Univ. Bourgogne Franche-Comté, INSERM, EFS BFC, UMR1098, Interactions Hôte-Greffon-Tumeur/Ingénierie Cellulaire et Génique, F-25000 Besançon, France; ^2^ DImaCell Platform, Univ. Bourgogne Franche-Comté, F-25000 Besançon, France; ^3^ EPIGENEXP Platform, Univ. Bourgogne Franche-Comté, F-25000 Besançon, France; ^4^ Tumorothèque de Besançon, Univ. Bourgogne Franche-Comté, F-25000 Besançon, France; ^5^ CHRU de Besançon, Univ. Bourgogne Franche-Comté, F-25000 Besançon, France; ^*^ These authors have contributed equally to this work

**Keywords:** breast cancer, autophagy, transcriptome, gene expression signature

## Abstract

Previous works have described that autophagy could be associated to both pro- and anti-cancer properties according to numerous factors, such as the gene considered, the step of autophagy involved or the cancer model used. These data might be explained by the fact that some autophagy-related genes may be involved in other cellular processes and therefore differently regulated according to the type or the grade of the tumor. Indeed, using different approaches of transcriptome analysis in breast cancers, and further confirmation using digital PCR, we identified a specific signature of autophagy gene expression associated to Luminal A or Triple Negative Breast Cancers (TNBC). Moreover, we confirmed that ATG5, an autophagy gene specifically expressed in TNBC, favored cell migration, whereas *BECN1*, an autophagy gene specifically associated with ER-positive breast cancers, induced opposite effects. We also showed that overall inhibition of autophagy promoted cell migration suggesting that the role of individual ATG genes in cancer phenotypes was not strictly dependent of their function during autophagy. Finally, our work led to the identification of *TXNIP1* as a potential biomarker associated to autophagy induction in breast cancers. This gene could become an essential tool to quantify autophagy levels in fixed biopsies, sort tumors according to their autophagy levels and determine the best therapeutic treatment.

## Introduction

According to the concept of autophagy network proposed by Behrends *et al.* in 2010, it has been described that autophagy, a lysosome-mediated degradation pathway, involves more than 40 ATG proteins (Autophagy-related proteins) and is regulated by more than 100 other proteins [1]. Autophagy regulates cell homeostasis and survival by controlling the degradation and recycling of protein aggregates, or organelles, in a multistep process. Each autophagy stage is associated to and mediated by specific proteins: *i*) Autophagy induction is driven by the core proteins ULK1 - ULK2 (Unc-51 Like Autophagy Activating Kinase 1 and 2), ATG11 and ATG13 and regulated by the mTOR-related proteins; *ii*) Autophagy initiation is directed by the core proteins UVRAG (UV Radiation Resistance Associated), VPS15, *BECN1* (BECLIN-1), VPS34, ATG2A/B, ATG9A/B, ATG14 and the WIPI family (WD Repeat Domain, Phosphoinositide Interacting); *iii*) Autophagy elongation is mainly regulated by the ATG4 family, ATG8 family, ATG3, ATG5, ATG7, ATG10, ATG12 and the ATG16L family); *iv*) Lysosome elongation is mediated by the LAMP family and V-ATPase and *v*) Fusion of the lysosome with the autophagosome is induced by the core proteins RAB38 and VAMP8. All these steps are regulated by dozen of kinases, GTPases, ligases and chaperon proteins. Besides the existence of a non-selective autophagy process during which the autophagosome randomly engulfs and degrades cell components, it has also been described the existence of a selective autophagy which is driven by specific adaptor proteins, such as SQSTM1 (Sequestosome 1)/P62, BNIP3L (BCL2 Interacting Protein 3 Like)/NIX, NBR1 (Neighbor Of BRCA1 Gene 1), ALFY (Autophagy-Linked FYVE Protein), PARKIN (Parkinson Protein 2, E3 Ubiquitin Protein Ligase) or PINK1 (PTEN Induced Kinase 1) to lead to the degradation of specific targets, such as ubiquitinated or aggregated proteins, damaged mitochondria, peroxisomes, and bacteria [2]. The role of autophagy in cancer is complex and paradoxical. On one hand, anti-tumor properties of autophagy have been associated to the first steps of cancer since it can prevent the transformation of healthy cells into cancer cells [3, 4]. On the other hand, autophagy could also favor tumor growth by conferring resistance to chemotherapies and promoting cell survival in a tumor microenvironment described to be poor in nutriments.

Breast Cancer (BC) is a heterogeneous disease with more than 2 million new cases each year in the world and with an incidence of 126 per 100,000 women in the USA. Despite increased therapy efficiency and earlier diagnosis, BC remains associated with a mortality rate of more than 22 per 100,000 women in this country. The usual molecular classification of BC is based on the quantification of the expression of main proteins, such as estrogen receptor (ER) (coded by the gene *ESR1*), progesterone receptor (PR), HER2 (human epidermal growth factor receptor 2) and KI67, a marker of proliferation. Consequently, four groups of BC have been described: Luminal A (ER^+^ or PR^+^, KI67-, HER2-), Luminal B (ER^+^ or PR^+^, KI67^+^ or HER2^+^), HER2 (ER-, HER2+) and triple negative (TNBC, ER-, PR-, HER2-) (for a review, see [5]). During the last decade or so, many studies demonstrated the major role of autophagy in Breast Cancer during tumor progression, tumor dormancy, metastasis progression or the apparition of resistance to treatments [6, 7].

The evaluation of autophagy in patient samples remains a challenging question due to the difficulty of quantifying a dynamic mechanism in non-living fixed tissues. Although autophagy was considered for decades as a dynamic protein process regulated by post-translational modifications, it appears now that many autophagy-related genes are regulated at the transcriptional level, as well. Regarding individual gene expression, a high expression of *MAP1LC3B* (usually called LC3B) has been associated to poor prognosis in triple negative breast cancer TNBC [8-10]. On the contrary, *BECN1* has been considered as a tumor suppressor gene [4, 11] and the expression of an ATG8 family member, *GABARAPL1*, has been associated to a good prognosis in BC [1-16]. These data therefore strongly suggest that proteins involved in different steps of autophagy (*e.g.* phagophore initiation or elongation) may present different and even opposite roles during breast tumorigenesis. An additional problem is the lack of knowledge on this matter since very little is known about the pattern of expression of autophagy-related genes in BC.

Altogether, these informations led us to hypothesize and demonstrate, using a metadata analysis of more than 150 autophagy-related genes followed by the biological confirmation of our Biocomputing data in a BC cohort, that BC subgroups are clearly associated to a specific autophagy transcriptional signature. Moreover, our results also unequivocally showed that different factors of the autophagy pathway, belonging to this signature, were directly involved in cancer aggressiveness and prognosis *via* both dependent and independent autophagy functions.

## RESULTS

**Figure 1:**
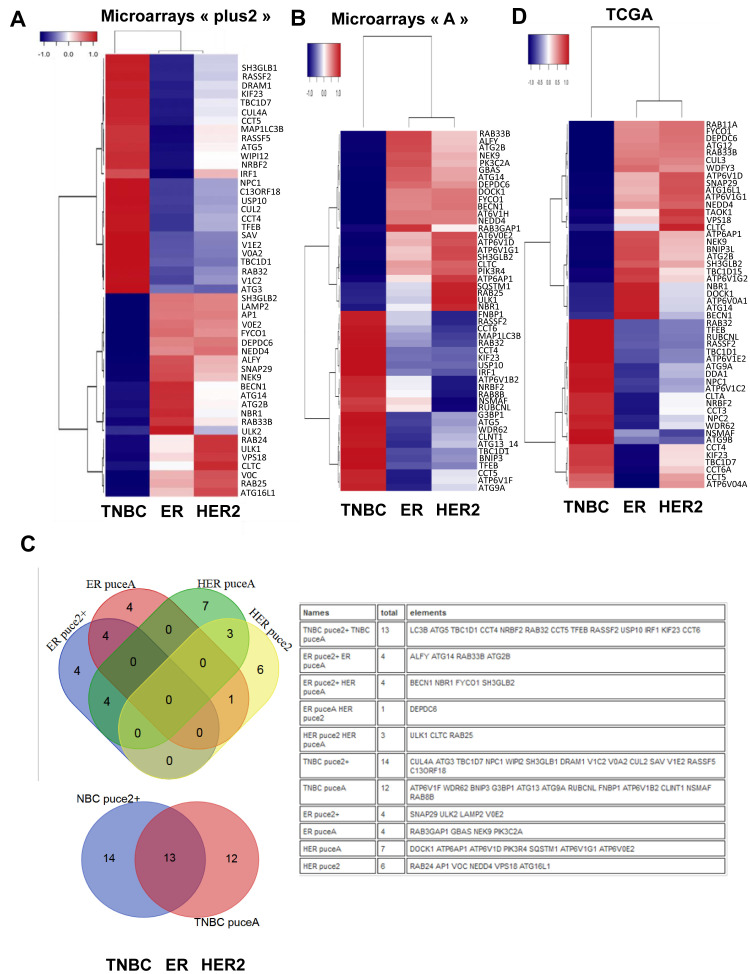
Breast cancer subgroups are associated with a specific autophagy gene expression signature. 2691 and 2806 samples, respectively, and the most variable autophagy genes were hierarchically classified according to correlations between gene expressions normalized from HG-U133 plus2 arrays **A.** or microarray “A” **B.** and BC groups. The color Dark red means relative high expression while the color Dark blue means relative low expression. **C.** Venn diagrams comparing the identified genes identified in HG-U133 plus2 arrays and microarrays in regards to BC subtypes. **D.** Heatmap representing the most variable autophagy genes described from the data extracted from 1022 normalized BC RNA-seq (TCGA).

Since, in previous studies, autophagy has been associated to both pro- and anti-cancer properties according to the cancer models used, we suggested that these differences could be linked to different and specific ATG gene expression. To answer this question, we first looked for a transcriptional autophagy signature in BC subgroups using 166 genes directly involved in autophagy, or associated to the autophagy network according to Behrends *et al.* [1] (Figure 1). We therefore used 5345 retrospective transcriptomes, obtained from raw data downloaded from public databases, which were then divided in 2 series corresponding to HG-U133A arrays and HG-U133 plus2 arrays. In each of the series, we counted more than 500 triple negative, 300 HER2 and 1800 Luminal tumors. The quantification of positivity or negativity of four genes (*ER, PGR, KI67, HER2*) produced robust classifications which were concordant with the Prediction Analysis of Microarray 50 (PAM50) classification based upon centroids [17]. A list of 50 genes related to autophagy and presenting the highest differential expression was used to build a heatmap. Our analysis clearly showed that *ATG* genes were differentially expressed between BC subgroups (Figure 1A). As expected, a similar profile was obtained between the HG-U133A arrays and the HG-U133 plus2 arrays (Figure 1B) confirming that a specific transcriptional autophagy signature was associated with BC subgroups. Venn diagrams confirmed that most of genes were similarly clusterized in both heatmaps (Figure 1C).

**Figure 2:**
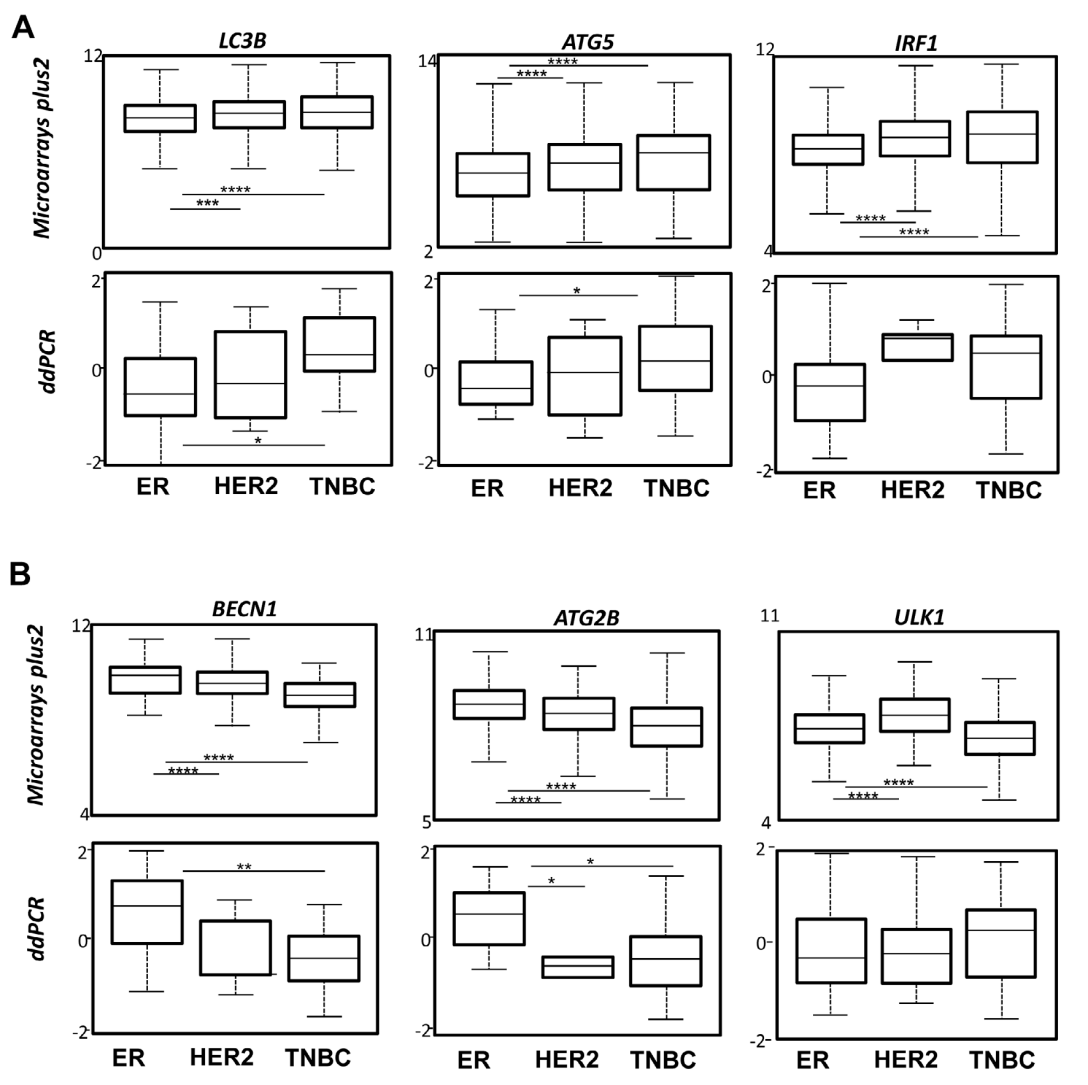
Confirmation of the specific autophagy gene expression signature using ddPCR in an independent cohort of BC. **A.** Expression quantification boxes built from the HG-U133 plus2 data for LC3B, *ATG5* and IRF1 gene expressions (top). Confirmation of the results obtained using ddPCR in an independent cohort (bottom). **B.** Expression quantification boxes built from the HG-U133 plus2 data for *BECN1*, *ATG2B* and ULK1 gene expressions (top). Confirmation of the results obtained using ddPCR in an independent cohort (bottom).

Amongst this signature, we selected 6 genes presenting the most important differences in microarrays plus2 (and confirmed in other transcriptome analyses) and which were representative of the BC subgroups: *IRF1, LC3B* and *ATG5* whose expression was increased in TNBC; *BECN1* and *ATG2B* whose expression was increased in the ER group (HER+ or -) and finally, *ULK1* whose expression was elevated in ER and HER2 groups compared to TNBC. To confirm whether our signature, and particularly our selection of genes, could be used as a prediction tool, we next quantified the expression of these 6 genes using the droplet digital PCR (ddPCR) technology in a new independent BC cohort. Amongst our 64 BC samples, 38 were qualified for ddPCR (with a housekeeping gene expression of *hsTBP* > 0.3 copies / µl). The BC subtypes were then classified using both IHC criteria (ER, PR and HER status) and the expression of *KI67* and *FOXA1* analyzed with the ddPCR (Supplemental Figure 1). Remarkably, the ddPCR data, obtained during the analysis of this new cohort, confirmed that *BECN1* and *ATG2B* expressions were the highest in ER tumors while *IRF1, LC3B* and *ATG5* expressions were increased in TNBC (Figure 2). The absence of confirmation of the association of *ULK1* expression with the HER subgroup using ddPCR was probably due to the low number of HER samples available.

**Figure 3:**
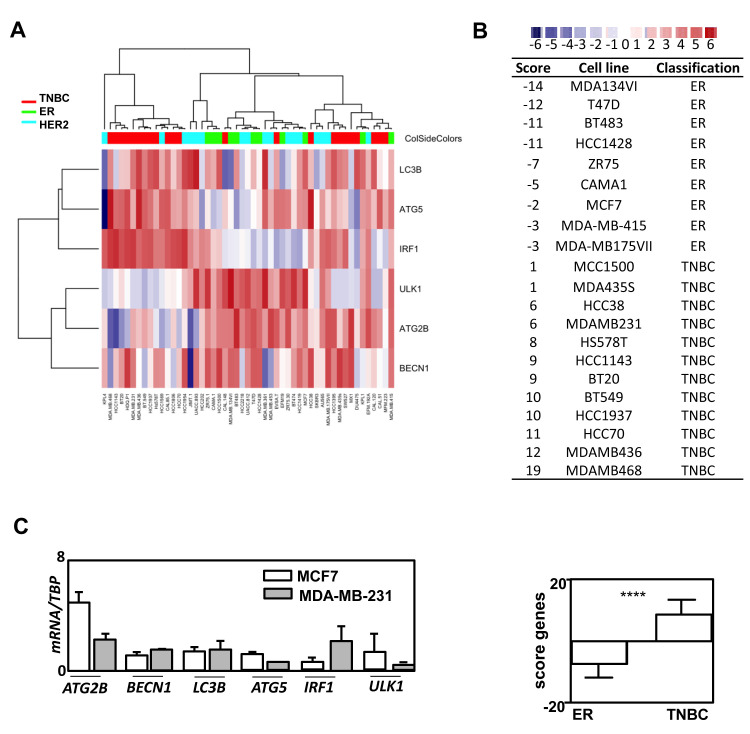
The restricted specific autophagy gene expression signature in BC groups efficiently discriminates BC cell lines. **A.** Heatmap representing the selected autophagy signature (*LC3B, *ATG5*, *IRF1*, ULK1, *ATG2B*, BECN1*) which was obtained from data of normalized HG-U133 plus2 with BC cell lines. **B.** The score of -6 to +6 according to heatmap intensity (from blue to red) was calculated as follow: values of *IRF1*, LC3B, *ATG5* minus values of *ATG2B*, BCN1. TNBC and ER cell lines were perfectly discriminated with this score (bottom). **C.** RT-qPCR confirmation of *ATG2B*, *BECN1*, LC3B, *ATG5*, *IRF1* and ULK1 expressions.

Since the role of autophagy in cancer has been frequently studied in BC cell models, we next compared the expression of autophagy genes in BC cells to our BC signature (Figure 3A). As expected, the autophagy profile of the model cell lines studied was strongly correlated to our transcriptional autophagy signature in regards of their BC classification [18]. For example, *IRF1, LC3B* and *ATG5* expressions were low while *ULK1, ATG2B, BECN1* expressions were elevated in ER-positive T47D cells whereas opposite results were observed for the TNBC MDA-MB-468 cell line. When we quantified gene expression in these cell lines (values of *IRF1, LC3B, ATG5* minus the values obtained for *ATG2B, BCN1*), the cell lines were perfectly discriminated between ER and TNBC subgroups (Figure 3B). Again, when cell lines were classified in regards to their LumA or TNBC status, the differences obtained were highly significant (*p* < 0.0001). Indeed, all negative scores corresponded to LumA cell lines whereas positive scores belonged to the TNBC group. Specific profiles of *ATG2B, BECN1* and *ATG5* expressions were then confirmed using qRT-PCR (Figure 3C) in MCF-7 and MDA-MB-231 cell lines, thus confirming the robustness of the heatmap data.

Since TNBC present a much more aggressive phenotype than ER-positive BC, we next wondered whether this transcriptional signature could be associated to good or bad prognosis. To do so, we used the KM-plotter database to analyze the survival rate of BC patients in regards to their expression of the 6 genes representative of our autophagy signature (Supplemental Figures 2-3). As expected and in agreement to our BC signature, a high expression of *BECN1, ATG2B* and *ULK1* was associated with a good prognosis, whereas the expression of *ATG5* was associated with a poor prognosis. No difference was observed for *LC3B* and, to our surprise, a high expression of *IRF1* was also associated to a good prognosis. Similarly, Kaplan-Meier analysis of other genes differentially expressed between TNBC and ER-positive BC (Figure 1) also determined the survival rate of patients. Indeed, a high expression of *ATG3, CCT4, CCT5* and *CCT6*, whose expression was also up-regulated in TNBC (Figure 1), were associated with a poor prognosis (Supplemental Figure 3), whereas a high expression of *ATG14* and *FYCO1*, whose expression was up-regulated in ER-positive BC (Figure 1), were associated with a good prognosis. We next used a TNBC gene signature/ER gene signature ratio to determine whether this value would further narrow the survival rate of patients. Our analysis showed that patients with a high *ATG5/BECN1* ratio indeed presented a lower survival rate (159.5 months) compared to the prediction using *BECN1* expression alone (185 months) or *ATG5* expression alone (189 months) (Supplemental Figure 4). A smaller difference was also observed with the *ATG5*/*ATG2B* ratio (75 months) compared to *ATG2B* expression alone (79.5 months). These data strongly suggested that our *ATG* signature could be used to predict BC prognosis.

When we looked at relapse-free survival at 5 years in an independent cohort using the ROC Plotter database [19], we observed that endocrine therapy, anti-HER therapy and chemotherapy were more efficient in ER, HER+ and TNBC cancers, respectively. No differences were observed for *IRF1* expression but we observed that a high *BECN1* expression was associated (*p* < 0.0001) with a higher response to endocrine therapy (Supplemental Figure 5) compared to the ER signature. A similar, but not significant tendency, was also observed for *ATG2B*. A high *ATG5* expression seemed inversely correlated to a response to endocrine therapy (*p* = 0.066) and to an anti-HER therapy (*p* < 0.01), but correlated to a significant response to chemotherapy according to the TNBC signature. Similarly, LC3B expression inversely correlated to both endocrine (*p* < 0.01) and anti-HER (*p* < 0.0001) therapy responses. Altogether, these data argued that our genes were specific markers of these BC subgroups but could also be used to predict prognosis, as well as treatment responses.

**Figure 4:**
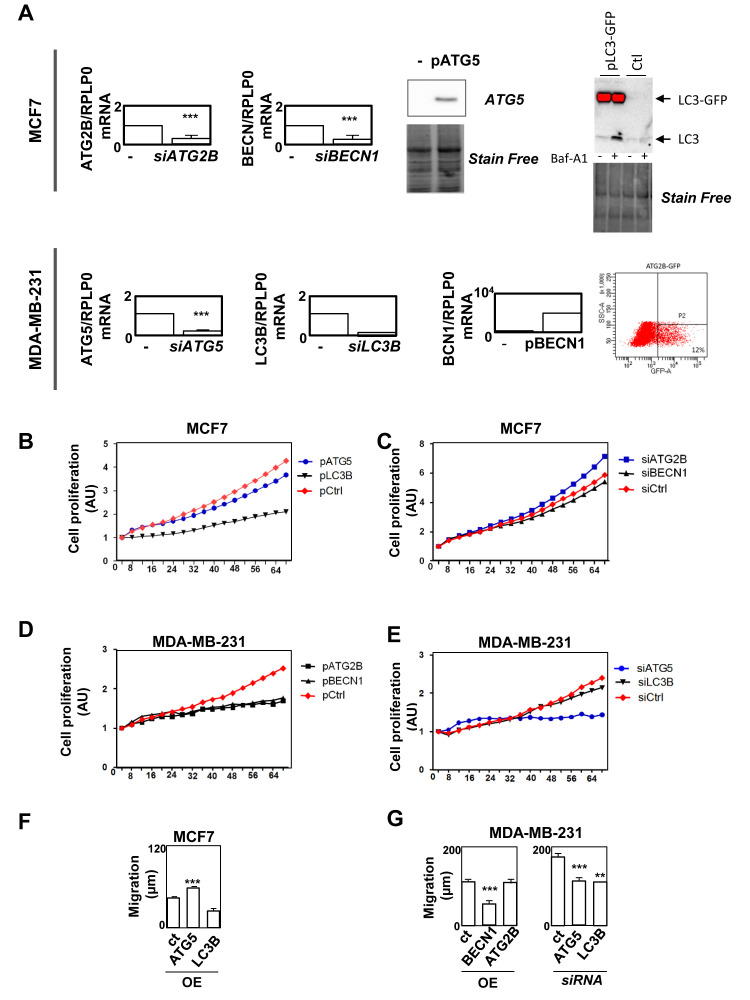
The modulation of gene expression of genes from the specific autophagy signature in BC groups affects cancer-related phenotypes. **A.** Overexpression or inhibition of LC3B, *ATG5*, *BECN1*, *ATG2B* expressions were confirmed using RT-qPCR, Western-blotting or flux cytometry analysis of MCF-7 or MDA-MB-231 cells. **B-E.** Cell proliferation monitored using the Incucyte technology in MCF-7 or MDA-MB-231 cells after overexpression or inhibition of the expression of the selected genes. **F-G.** Cell migration measured using a wound healing assay after 8 h in MCF-7 or MDA-MB-231 cells after overexpression or inhibition of the expression of the selected genes.

Since our gene signature was associated to prognosis and that these genes all belonged to the autophagy process, we next asked whether they were directly involved in cancer-related phenotypes. We decided to focus our analysis on *ATG5*, LC3B, *ATG2B* and *BECN1* genes because they strongly discriminated ER and TNBC BC (Figures 1 and 2). We therefore overexpressed (OE) or silenced the expression of these genes in BC models, ER-positive MCF-7 and TNBC MDA-MB-231 cell lines, using transfection of plasmids or siRNA. *ATG2B*, whose expression was increased in LumA, and LC3B, whose expression was increased in TNBC, were selected accordingly to our BC signature (Figure 4A). Surprisingly, overexpression of LC3B reduced MCF-7 cell proliferation but as described above, although LC3B expression was found associated with TNBC in our heatmap, this gene was not discriminant for prognosis (Supplemental Figure 3). No significant effects were observed following overexpression of *ATG5* or inhibition of *ATG2B* or *BECN1* expression in these cells (Figure 4B-4C). Regarding the MDA-MB-231 cells, in agreement with our BC signature, overexpression of *BECN1* or *ATG2B* decreased cell proliferation whereas inhibition of *ATG5*, but not LC3B, also reduced cell proliferation in this TNBC cell model (Figure 4D-4E). We next measured cell migration which is a feature much more representative of cancer cell aggressiveness than *in vitro* cell proliferation. In MCF-7, overexpression of *ATG5*, but not LC3B, increased cell migration (Figure 4F). In MDA-MB-231 cells, which present a higher basal migration rate, inhibition of *ATG5* expression decreased cell migration but inhibition of *ATG5* or LC3B expression reduced cell migration, as well (Figure 4F). Taken together, these data strongly suggested that autophagy genes, linked to our BC signature, were, at least partially, involved in cancer aggressiveness.

**Figure 5:**
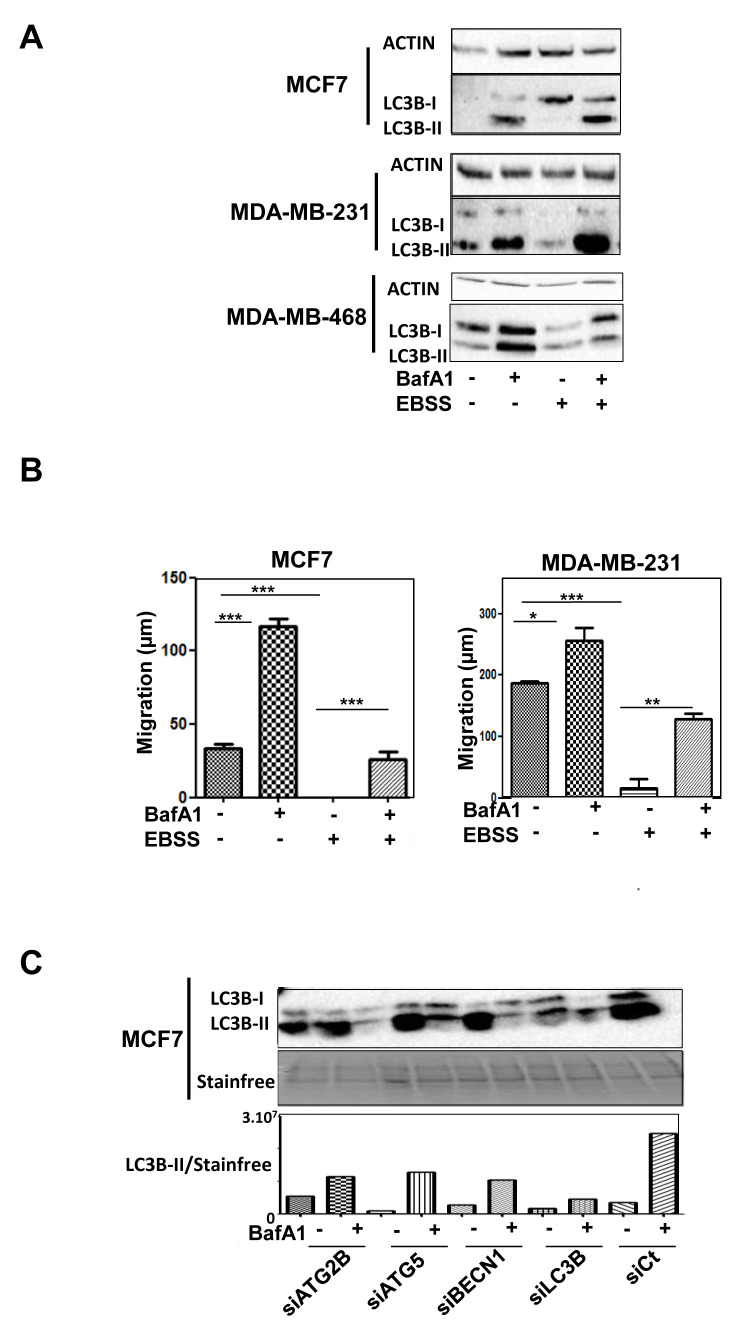
Autophagy directly affects cancer-related phenotypes. **A.** Efficient basal autophagy and EBSS-induced autophagy weres confirmed using WB (LC3B-II accumulation) in both MCF-7 and MDA-MB-231 cells. **B.** Cell migration measured using a wound healing assay after 8 h in MCF-7 or MDA-MB-231 cells, previously treated, or not, with BafA1 (autophagy blockage) and/or EBSS (autophagy induction). **C.** Effect of the inhibition of the expression of the selected genes on autophagy flux.

Nevertheless, we hypothesized that apparent contradictory results may be explained by their specific role in the autophagy process or by autophagy-independent functions. We therefore used MCF-7 and MDA-MB-231 cell lines, which both presented a high basal autophagy flux, quantified by the measure of LC3B-II accumulation following BafA1 or EBSS treatment, compared to other cells lines such as MDA-MB-468 which is, for example, not inducible by EBSS (Figure 5A). The blockage of autophagy by BafA1 increased cell migration, whereas an induction of autophagy by EBSS strongly inhibited cell migration in these cell lines (Figure 5B). A combination of EBSS and BafA1 partially restored cell migration. These data strongly supported the idea that autophagy inhibited cell migration in both cell lines. We next analyzed the effects of the modulation of *ATG5*, *BECN1*, *ATG2B* and LC3B genes on autophagy levels. Interestingly, an inhibition of the expression of these 4 genes dramatically decreased autophagy flux (Figure 5C). Combined together, all these data argued that autophagy decreased cell migration in BC cell lines and that the autophagy genes specifically associated to BC subgroups exerted their pro- and anti-cancer properties in both an autophagy-dependent and independent manner.

**Figure 6:**
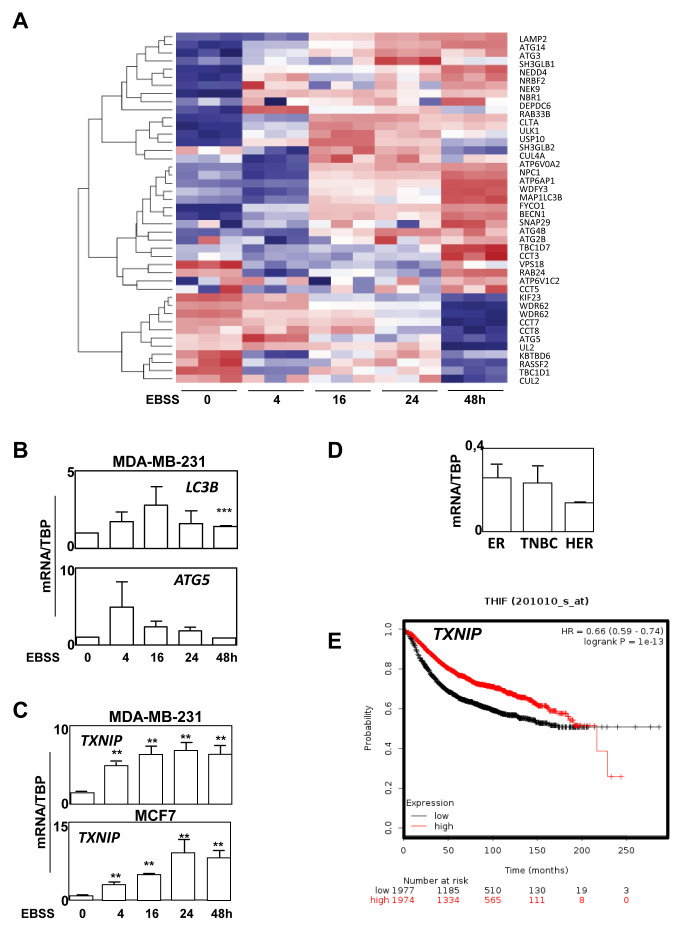
ATG BC signature and TXNIP expressions are markers of autophagy induction. **A.** Heatmap with the 40 most affected ATG-related genes in MDA-MB-231 cells treated with EBSS for 0 to 48 h to induce autophagy. **B-E.** B. Modulation of mRNA expression level of LC3B and *ATG5* during autophagy in MDA-MB-231. C. A progressive increase in TXNIP expression was confirmed using RT-qPCR in MCF7 and MDA-MB-231. D. mRNA expression level of TXNIP demonstrating than TXNIP expression is independent of BC subgroup. E. Kaplan Meier data analysis revealed a significant better prognosis for patients presenting a high TXNIP expression.

We next wondered whether ATG expression could also be used to evaluate autophagy both in fixed cancer biopsies. Since autophagy modulation may be a useful tool to regulate BC growth or progression, it would be important to better evaluate autophagy levels in tumors in order to develop and provide personalized therapies. However, autophagy is a dynamic flux process, and it is difficult to evaluate the level of autophagy in patients from the analysis of fixed BC biopsies. Indeed, a high level of LC3B-II may be a sign of a strong induction of autophagy, related to a high production of autophagosomes, as well as a blockage of the autophagy flux, linked to a loss of an efficient elimination of autophagosomes by lysosomes leading to their accumulation. We therefore asked whether ATG expression and our BC signature would help to determine the levels of autophagy flux. To do so, we decided to look for a transcriptional signature of the induction of autophagy and we performed RNA-seq experiments using MDA-MB-231 (TNBC) cells treated with EBSS in order to induce autophagy for 4 to 48 h. A heatmap of the most differentially expressed ATG genes is presented in Figure 6A. Interestingly, when ATG genes were classified using a heatmap, it appeared that *ATG5* expression increased after 4 h of EBSS treatment and then progressively decreased during 48 h. *ATG2B*, *BECN1* and LC3B expression were also increased during the treatment. According to our BC autophagy gene signature, these data suggested that autophagy might be more induced in ER-positive BC than in TNBC. It is noteworthy that the expression profiles of LC3B and *ATG5* were then confirmed using qRT-PCR following autophagy induction with EBSS (Figure 6B). However, since these genes belonged to our BC signature and were associated to specific BC groups, we also searched, in our total transcriptome, for an independent gene which could be added to our signature to evaluate autophagy induction independently of the BC subgroup. Following this research, we selected TXNIP whose expression appeared strongly induced after EBSS treatment in our 2 independent RNA-seq analyses (data not shown), and which appeared to be one of the most robust differentially expressed genes. We also confirmed this important increase of TXNIP expression using qRT-PCR in both MCF-7 and MDA-MB-231 cells (Figure 6C), and therefore demonstrated that TXNIP expression was not related to ER-positive or TNBC BC (Figure 6D).

As expected, a KM-plotter analysis revealed that patients presenting a high expression of TXNIP were associated with the best prognosis (*p* = 1E-13) (Figure 6E). Interestingly, when TXNIP expression was independently analyzed in ER-positive BC or in TNBC subgroups, this biomarker remained discriminant for prognosis showing that this gene could be a good marker of autophagy induction (Supplemental Figure 6B-6C). Similarly, using ROC plotter, a high expression of TXNIP was associated with a significant higher response to any chemotherapy (*p* = 3.2E-2) and seemed to be also associated with a higher response to endocrine therapy (*p* = 0.08) confirming that TXNIP expression was associated with a good prognosis, independently of the grade of the BC (Supplemental Figure 6C). On the opposite, although LC3B, *ATG5*, *ATG2B* and *BECN1* expressions were strongly associated to a specific BC group and that these genes regulated cell migration, none of these genes alone was discriminant for RFS in ER-positive BC or in TNBC subgroups. Our data therefore clearly showed that the quantification of *ATG5*, LC3B, *ATG2B*, *BECN1* and TXNIP expressions would help to better discriminate and classify BC tumors.

## DISCUSSION

Since autophagy is a complex mechanism previously associated with both pro- and anti-tumor properties, we decided to analyze the expression of more than 150 autophagy-related genes in a meta-analysis of BC transcriptomes to avoid bias of selection which could explain apparent contradictory published data. Interestingly, transcript levels of autophagy genes were not evenly distributed amongst the different tumor subtypes and we identified a transcriptional autophagy signature (*ATG5*, LC3B, *ATG2B*, *BECN1*, *IRF1*) of BC concordant in microarrays A, microarrays plus2, RNA-seq TCGA and this signature was also confirmed in a small independent cohort using ddPCR.

Amongst the genes found in our transcriptional autophagy signature, *BECN1* expression has already been shown to be modified in BC. Indeed, according to previous reports describing *BECN1* as a tumor suppressor gene, we found that *BECN1* expression was correlated with the ER-positive group and that overexpression of this gene reduced both cell proliferation and migration [4, 11, 20]. These data were also in agreement with previous works showing that *BECN1* expression was inversely correlated to the BC grade and that ER-negative BC were associated with low *BECN1* expression [21] [22]. These data, and our transcriptional signature in BC, strongly suggested that proteins involved in autophagy induction were protective against cancer phenotypes. In agreement with our observations, a meta-analysis using 23 independent studies recently concluded that a high *BECN1* expression was a favorable predictive factor for overall survival (OS) in BC, gastric cancers and lymphomas, whereas high levels of LC3B were inversely correlated with OS in BC [23]. Very little is known about the role of *ATG2B* in cancer, but a decreased expression of this gene linked to DNA methylation in invasive ductal carcinomas might participate in BC tumorigenesis [24]. Moreover, frequent mutations in the *ATG2B* gene were also reported in other cancers [25].

On the opposite, our autophagy signature was associated with an increased expression of LC3B, ATG3 and *ATG5* (and CCT4-6) in TNBC suggesting that proteins associated to the elongation step of autophagy were in favor of tumorigenesis (Supplemental Figure 7). How these differences could be involved in tumorigenesis and how the expression of these genes could be linked to ER expression remain unknown and should be explored in the future. We know that the expression of LC3B expression, at both mRNA and protein levels, has been previously associated to TNBC [9, 26] but different studies reported contradictory results regarding *ATG5* expression [27, 28]. Indeed, a high *ATG5* expression measured by IHC has been associated with disease free survival in BC [28], whereas a high *ATG5* mRNA expression was correlated with shorter OS in AML [29]. Similarly, expression of both LC3B and *ATG5* were promoted by hypoxia in a HIF-1-dependent manner in gliomas or prostate cancer cells [30, 31]. Moreover, inhibition of *ATG5* expression in glioma cell models strongly reduced cell mobility with increased chemosensitivity under hypoxia suggesting that this gene was also associated with aggressiveness in different cancer models. *IRF1* was very recently associated to mesenchymal features, basal like and CLAUDIN-low BC [32], data which are in agreement with our results showing that this gene was associated to TNBC. *IRF1* regulates autophagy induction but is not directly involved in the autophagy process, and it participates in numerous pathways which may explain why its expression is associated with a good prognosis in BC patients despite its association with TNBC. These data were also supported by the observation reporting that an induction of *IRF1* expression in BC cells provoked apoptotic cell death [33]. Indeed, although some of these genes have previously been associated to BC, to our knowledge, none of these previous reports used metadata analysis including several thousands of patients and 4 independent analyses. Altogether, these data strongly showed that our BC signature using the systematic quantification of *ATG2B*, *BECN1*, *LC3B*, *ATG5*, and eventually *ULK1* and *IRF1* led to a robust discrimination of BC subgroups and helped for prognosis.

Since autophagy is a dynamic process formed by the formation and the degradation of autophagosomes, induction of autophagy as well as disruption of the autophagy flux may both provoke autophagosome and ATG protein accumulation leading to a difficult quantification of the autophagy flux in fixed biopsies by IHC. Therefore, a better understanding of autophagy mechanisms is a major point to develop future personalized therapies based on autophagy-targeting drugs. Quantification of autophagy gene expressions could therefore be appropriate to determine autophagy levels in biopsy samples. We described that the quantification of *ATG2B*, *ATG5*, *LC3B* expressions would help to determine autophagy levels, but since these genes were associated to a specific BC group, we also looked for an independent marker. Amongst the genes whose expression was the most increased following autophagy induction in cells treated by EBSS (Figure 6A), *TXNIP* appeared as a good candidate for a biomarker of autophagy induction. Even if the protein TXNIP is not directly involved in autophagy flux, the regulation of its expression is, at least partly, independent of autophagosome number and may well reflect autophagy levels. Indeed, this protein has previously been associated to autophagy by Shui Qiao *et al.* who reported a direct role of TXNIP in the regulation of ATG4B activity as well as autophagy induction [34]. Moreover, an increase in *TXNIP* expression was progressive (Figure 6B-6C) suggesting that a correlation could be established between the level of autophagy and the level of *TXNIP* expression. Based on *TXNIP* expression and the fact that a blockage of autophagy promoted cell migration (Figure 5), our data argued that autophagy induction and initiation limited aggressiveness but that individual ATG genes, such as *ATG5* and *LC3B*, might promote cancer-related phenotypes in a possible autophagy-independent manner.

In conclusion, our work demonstrated, for the first time, that the quantification of specific autophagy genes, such as *BECN1*, *ATG5*, *LC3B*, *ATG2B* and *TXNIP*, could help in establishing a new BC stratification in the future and developing new autophagy-linked therapies.

## MATERIALS AND METHODS

### Patients

Human samples were collected according to French laws and the recommendations of the French National Committee of Ethics. This study has been approved by the scientific committee of “the Tumorothèque Régionale de Franche-Comté BB-0033-00024”. All human samples were collected by the Hospital of Besançon (France) at the “Tumorothèque Régionale de Franche-Comté BB-0033-00024”. Collection of samples and their use (AC-2010-1163) for studies have been approved by the French “Ministère de la Recherche” and by the CPP EST II. We obtained all necessary consents from any patients involved in the study.

### Cell culture

MCF-7 and MDA-MB-231 cells were respectively cultured in DMEM low glucose (Dutscher) supplemented with 5 or 10% SVF (Dutscher) and 1% penicillin-streptomycin (Dutscher) with 5% CO2 in an atmosphere saturated in humidity. Plasmids for overexpression of *BECN1* (gift from Dr G. Kroemer), GFP-LC3B (gift from Dr. Elazar, The Weizmann Institute of Science, Rehovot, Israel), GFP-*ATG2B* (gift from Dr Li Yu [35]), *ATG5* (#24922, addgene) or empty vector were transfected using JetPrime reagent (114, Polyplus) according to the manufacturer’s instructions. The inhibition of gene expression was performed using siRNA (Eurogentec) and Interferin (409, Polyplus) according to the manufacturer’s recommendations. When indicated, cells were treated with EBSS (E3024, Invitrogen) for 2 to 24 h or 500 nM BafA1 (B1793, Sigma-Aldrich) for 2 h.

### Cell proliferation and wound healing assay

Cell proliferation was analyzed using the Incucyte automatic microscope (Sartorius) following cell seeding (4,000 for MCF-7 and 2,000 for MDA-MB-231). Migration assays were performed using culture-insert 2 wells in 35 mm microdishes (81176, Ibidi). 30,000 pretreated cells were plated in each well and the insert was removed 24 h later. Migration was monitored for 8 h.

### Western-blotting

Total protein extracts were obtained from scraped cells which were harvested and lysed for 30 min on ice in RIPA buffer (50 mM Tris-HCl, pH 8, 150 mM NaCl, 1% Triton X100, 0.5% DOCA, 0.1% SDS) supplemented with protease inhibitors (104 mM AEBSF, 1.5 mM pepstatin A, 1.4 mM E-64, 4 mM bestatin, 2 mM leupeptin, 80 µM aprotinin). 20-40 µg of total proteins were separated on TGX acrylamide gels (1610172, Biorad) at 280 V for about 30 min using Biorad Protean III system and transferred onto PVDF (1704157, Bio-Rad) membranes for 10 min with the Transblot turbo (1704150, Biorad) according to the manufacturer’s recommendations. Membranes were saturated in 0.1% TBS-Tween 20 and 5% milk for 1 h and then incubated with primary antibodies anti-LC3B (L8918, Sigma-Aldrich), anti-ACTIN (A5060, Sigma-Aldrich) overnight at 4°C. Membranes were washed 3 times with TBS-Tween 20 0.1%, incubated with secondary anti-rabbit HRP conjugate antibodies according to manufacturer’s instructions (P.A.R.I.S.). (BI2407, BI2413C, P.A.R.I.S.). Membranes were washed 3 times with TBS-Tween 20 0.1% and incubated with Clarity Western Cl substrate (1705051, Biorad) and chemiluminescence was monitored using a Biorad ChemiDoc™XRS+.

### Transcriptomes, qRT-PCR and Droplet Digital RT-PCR

RNA was isolated from cells using the Tri Reagent (TR118, Molecular Research Center) as recommended by the manufacturer and as previously described [36]. Reverse transcription was performed using 12 U M-MLV reverse transcriptase (M-1302, Sigma-Aldrich), 0.25 µM oligodT (Eurogentec), 1.25 µM random hexamers (C118A, Promega) and 1.5 µg total RNA according to the manufacturer’s instructions (Sigma-Aldrich). Primers (Eurogentec) were designed using the primer 3 software: *ULK1*: 5’-CAGACAGCCTGATGTGCAGT-3’ and 5’-TCAATGCGCTGGTAGTTCTG-3’, *LC3B*: 5’-TTGAGCTGTAAGCGCCTTCT-3’ and 5’-AGCAGCATCCAACCAAAATC-3’, *ATG5*: 5’-AGGATCAGAATGCAGGGAAC-3’ and 5’-AAGATCAGAATGCAGGGAAC-3’, *IRF1*: 5’-GAAACTGGATGGCAAGTGCT-3’ and 5’-CAGAGATGCTGCTCCAAAAA-3’, *ATG2B*: 5’-GTGCTGCTACCCGGGACATTA-3’ and 5’-TGCCATCTGCTGCATTTCAAG-3’, *BECN1*: 5’-TCACCATCCAGGAACTCACA-3’ and 5’-CCTGGCGAGGAGTTTCAATA-3’, TBP: 5’-CACGAACCACGGCACTGATT-3’ and 5’-TTTTCTTGCTGCCAGTCTGGAC-‘3, Ki67: 5’-ATTGAACCTGCGGAAGAGCTGA-3’ and 5’-GGAGCGCAGGGATATTCCCTTA-3’, *FOXA1*: 5’-CCCCTTTGTCCTCTCTACCC-3’ and 5’-GACATGACCATGGCACTCTG-3’. Absolute quantification of cDNA was performed using the QX200 Droplet Digital PCR system (ddPCR, Biorad) with the QX200 ddPCR EvaGreen Supermix (1864033, Biorad) according to the manufacturer’s instructions. Absolute quantification was performed using the QuantaSoft™ Software (BioRad). Quantitative PCR (qPCR) were performed as duplicates using the Step one plus Real-Time PCR system (Applied Biosystems, France) and the Premix Ex Taq™ DNA Polymerase kit (RR039B, Takara) according to the manufacturer’s recommendations. RNA-seq was performed in MDA-MB-231 cells by Integragen society.

### Statistics

Two distinct Affymetrix series were built following GC-RMA normalization: one from HG-U133A arrays (n = 2806) and one from HG-U133 plus2 arrays (n = 2691) in which 6500 additional probes were added. Each serie was standardized using the Aroma R package which was developed to normalize multicenter extremely large Affymetrix data sets. The same computations were performed in the two data sets in order to cross-validate the results. Samples were classified with a reduced number of genes (as described in the St Gallen classification [37-39]) and with classifiers using 50 or more than 300 genes (PAM50, CIT and IntClust centroids). Transcriptome analysis was performed using the R software version 3.3.1 from the R Foundation for Statistical Computing (Vienna, Austria). Transcriptomes were analyzed from Affymetrix series (GPL570 platform) after GC-RMA normalization. 2830 samples were used from the following GEO series: GSE12276, GSE12790, GSE18931, GSE2109, GSE22035, GSE23720, GSE23994, GSE25407, GSE26910, GSE30010, GSE3744, GSE5764, GSE17700, GSE26639, GSE16446, GSE18864, GSE22513, GSE19615, GSE20685, GSE21653, GSE23177, GSE9195, GSE6532. Samples were classified using the PAM50 method using 50 genes (Genefu R package). For RNA-seq, TCGA data analysis was performed using 1222 samples. Differences between means for each cell experiment were performed using the t-test and the GraphPad software. The expression score was calculated as follow: sum of (individual score values from *IRF1*, *LC3B*, *ATG5*) minus (individual score values of *ATG2B*, *BCN1*). Each individual score value was calculated from -6 to 6 depending of the level of intensity.

## SUPPLEMENTARY MATERIALS AND FIGURES



## Abbreviations

BL-1/2: basal-like
BC: breast cancer
LumA/B: luminal A or B
BafA1: bafilomycin A1
TNBC: triple negative breast cancer
ULK1 and 2: Unc-51 Like Autophagy Activating Kinase 1 and 2
